# Association of Sleep and Nap Duration With Cardiovascular Disease-Free Life Expectancy Among Older Adults

**DOI:** 10.1093/geroni/igaf122.768

**Published:** 2025-12-31

**Authors:** Ruoxuan Rosalyn Li, Abhijit Visaria, Stefan Ma, Rahul Malhotra

**Affiliations:** Duke-NUS Medical School, Singapore, Singapore; Duke-NUS Medical School, Singapore, Singapore; Singapore Ministry of Health, Singapore, Singapore; Duke-NUS Medical School, Singapore, Singapore

## Abstract

Sleep and nap durations have been linked with many health outcomes, yet their impact on health expectancy remains understudied. We investigated the association of nocturnal sleep and daytime nap durations with total life expectancy (TLE), and life expectancy (LE) with and without cardiovascular diseases (CVD), among older adults in Singapore. Representative longitudinal data (three waves; 2009-2015) of community-dwelling Singapore residents aged ≥60 years (n = 3452) was used. We applied multistate life table methods to estimate TLE, and LE with and without CVD, by sleep duration and nap duration, adjusting for a range of socio-demographic and health variables. At ages 60, 70, and 80 years, there was no difference in TLE or LE without CVD among those with short, recommended and long sleep. However, long (versus recommended) sleep was associated with a shorter LE with CVD. At ages 60 and 70, the proportion of remaining life without CVD was higher, and with CVD was lower, among those with long (versus recommended) sleep. At ages 60, 70, and 80 years, long nap was associated with a shorter TLE and LE without CVD, versus none or short nap. LE with CVD or proportion of remaining life with and without CVD did not differ by nap duration. Our findings reflect the complex relationship between sleep and nap durations and life and health expectancy outcomes. Further research is needed to elucidate underlying mechanisms and guide recommendations for optimal nocturnal sleep and daytime nap durations in older adults.

